# Reference ranges for complete blood count in children and adolescents with Down syndrome

**DOI:** 10.3389/fped.2024.1510733

**Published:** 2024-12-11

**Authors:** Martina Lattuada, Giulia Capitoli, Marco Casati, Alessandra Lazzerotti, Oscar Maglia, Giulia Maria Ferrari, Chiara Fossati, Andrea Biondi, Alessandro Cattoni, Paola Corti

**Affiliations:** ^1^ School of Medicine and Surgery, University of Milano-Bicocca, Monza, Italy; ^2^ Department of Medicine and Surgery, Bicocca Bioinformatics Biostatistics and Bioimaging Research Centre-B4, University of Milano-Bicocca, Monza, Italy; ^3^ Biostatistics and Clinical Epidemiology, Fondazione IRCCS San Gerardo dei Tintori, Monza, Italy; ^4^ Clinical Chemistry Laboratory Medicine, Fondazione IRCCS San Gerardo dei Tintori, Monza, Italy; ^5^ Pediatrics, Fondazione IRCCS San Gerardo dei Tintori, Monza, Italy; ^6^ Tettamanti Center, Fondazione IRCCS San Gerardo dei Tintori, Monza, Italy

**Keywords:** Down syndrome, hematopoiesis, complete blood count, reference ranges, pediatric

## Abstract

**Introduction:**

Down syndrome (DS) is linked to unique hematopoietic characteristics that affect complete blood count (CBC) parameters. Accurate reference ranges are essential for proper CBC interpretation in this population.

**Methods:**

This retrospective study analyzed 2,627 CBCs from 481 DS patients, aged 31 days to 18 years, at a tertiary care center in Italy. Patients with significant comorbidities were excluded to ensure a homogeneous group.

**Results:**

CBC parameters were assessed to establish age- and sex-specific reference ranges. Centile charts were developed for each parameter, and an online tool was created to allow clinicians to compare individual CBC results with the new ranges. Comparisons with the general pediatric population revealed significant differences, particularly in hemoglobin, hematocrit, and mean corpuscular volume, which were higher in DS (*p* < 0.001). In contrast, a significant percentage of CBCs showed white blood cell counts below the 2.5th centile of healthy controls (*p* < 0.001), except for the 31 days–1 year age group. A similar trend was observed for lymphocytes (*p* < 0.001) in the 1-18 years group.

**Discussion:**

These newly established DS-specific reference ranges provide clinicians with a crucial tool for evaluating CBC results, potentially reducing unnecessary tests and emphasizing the need for tailored clinical assessment in managing this unique population.

## Introduction

Down syndrome (DS) is the most common chromosomal aneuploidy among live births and the most frequent cause of intellectual disability related to a demonstrable microscopic chromosomal aberration (1), with an incidence of 1:700–1,000 live births and a prevalence of 1:400–3,000 in the general population (2). From a hematological perspective, patients with trisomy 21 may exhibit alterations in all hematopoietic lineages, as a consequence of the overexpression of several genes involved in the regulation of the hematopoietic system (3). In fetuses and neonates with DS, studies on hematopoiesis have shown a marked expansion of megakaryocytic and erythroid progenitors, alterations in myeloid progenitors and severe impairment in lymphocyte development (4–6). These alterations appear to persist throughout infancy, but systematic studies have not yet been conducted to confirm this assumption (6). Accordingly, newborns with DS may present with a wide spectrum of hematological disorders, including polycythemia, thrombocytopenia, neutrophilia, and lymphocytopenia (7–10). Conversely, macrocytosis and leukopenia are findings more commonly recorded among children, adolescents and adults (11–15). Macrocytosis is often regarded as the result of an increased expression of the cystathionine beta-synthase gene (CBS), leading to an increase in folate re-methylation pathways and, subsequently, to a prolonged cell cycle and increased red blood cell volume (12). Complete blood count (CBC) abnormalities, in most cases, are not associated to any other underlying pathological condition and have no clinical repercussions. Conversely, DNA mutations occurring in the setting of increased cellular proliferation in hematopoietic sites have been extensively described in children with DS, leading to pathological phenomena ranging from transient abnormal myelopoiesis in newborns (prevalence: 4%–10%) (16) to acute leukemia in infants, children and adolescents (prevalence: 3%) (17).

Given the above-mentioned specificities of children with DS, the development of syndrome-specific CBC reference ranges would prevent clinicians from labelling normal findings as pathological and, accordingly, from prescribing unnecessary biochemical monitoring. Conversely, reference values would promote timely detection of the few abnormalities deserving prompt additional assessment.

To the best of our knowledge, only two published studies have reported the distribution of CBC parameters in pediatric cohorts of individuals with DS (18, 19). As peripheral blood counts show remarkable changes along the maturation process that leads from fetal to adult erythropoiesis, the interpretation of CBC parameters is based on the comparison with reference ranges of normality drawn from the otherwise healthy pediatric population (20). The aim of this study is to outline the normal distribution of CBC values in pediatric subjects with DS, in order to identify the age- and gender-specific reference ranges for each hematological parameter and to compare them with those published for the general pediatric population (21, 22).

## Methods

### Data sources and patients

We designed a retrospective, observational, monocentric analysis. Eligible patients were retrieved from the pediatric outpatient Clinic of Fondazione IRCCS San Gerardo dei Tintori Hospital, Monza (Italy).

We included children and adolescents diagnosed with DS, aged between 31 days and 18 years, of both sexes and with different ethnical background. Only CBCs withdrawn in otherwise healthy children, on an outpatient basis and as a part of routine follow-up were gathered. We excluded CBCs of patients diagnosed with underlying clinical conditions, i.e., hematological or oncological diseases, cyanogenic congenital heart diseases, severe/chronic lung diseases, and severe obstructive sleep apnea syndrome. Also, CBCs assessed in the setting of infectious events, acute illnesses, emergency or following transfusional events or recent surgery were excluded. Finally, we ruled out patients treated with ongoing myelosuppressive medications. The blood counts of DS subjects with celiac disease were included in the analysis only if performed after 12 months of a gluten-free diet.

The study was conducted in accordance with the Declaration of Helsinki. Informed consent was obtained from patients and their families, and the investigation was approved by the local ethical board. All data were anonymized.

### Hematological parameters

In our institution, CBC is performed annually in pediatric patients diagnosed with DS, in accordance with the guidelines of American Academy of Pediatrics (AAP). CBCs were performed between 2004 and 2023 by two different hematological analyzers: Sysmex XN-10 and Beckman Coulter DxH800. The following parameters were retrieved: red blood cells (RBC, ×10^12^/L), hemoglobin (HB, g/L), mean corpuscular volume (MCV, fl), hematocrit (HCT, %), white blood cells (WBC, ×10^9^/L), absolute neutrophil count (NEU, ×10^9^/L), absolute lymphocyte count (LYM, ×10^9^/L), and platelets (PLT, ×10^9^/L).

### Statistical analyses

CBC parameters assessed by two different lab analyzers were compared by non parametric regression model and reported as time trendlines by cubic splines. Mean (SD), or median (interquartile ranges, IQR) were used to describe continuous variables and frequencies (percentages) for qualitative variables. Age and genders-specific CBC parameters reference ranges were estimated via regression quantiles. The lower and the upper limits of each Gaussian distribution was referred with reference to the 2.5th and 97.5th percentiles, respectively. Inequality constraints were used to ensure both monotonicity and non-crossing of the estimated quantile curves, and penalized splines were employed to model the nonlinear patterns concerning age. The distribution of each blood count parameter was graphically delineated with sex-specific nomograms, displaying the 2.5th, 10th, 25th, 50th, 75th, 90th, and 97.5th percentiles of the recorded age-specific data. Age groups were defined for each parameter, in order to establish reference ranges and compare them with those of the general pediatric population (21, 22) and with two recent studies conducted on children with DS (18, 19). A one-sample test on proportion was used to compare reference ranges of our patients with DS and healthy controls (21, 22) with respect to specific reference thresholds. The tests were two-sided, and the significance level was set at.05. Wilcoxon-test was used to compare CBCs distribution, by age groups and sexes, of our study with those of the healthy control population and DS patients from Harvey et al. (19) and those of DS patients from Garcia de la Puente et al. (18); the results were also graphically represented. The significance level used to reject the null hypothesis is 0.05. All the statistical analyses were performed with the opensource R software v.4.4.2.

## Results

### Study population

Four-hundred-eighty-one patients out of the 559 eligible from our Institution met the criteria for inclusion in the study. Overall, 2,627 CBCs were collected. For this cohort, the average number of CBCs for each patient was found to be 5.67 ± 3.7 SD. Of the 481 patients enrolled, 249 were males (51.8%). From an ethnical perspective, 438 (91.1%) were Caucasian, 20 (4.2%) originating from North Africa, 8 (1.6%) from Central Africa, 8 (1.6%) from Southeast Asia, 6 (1.2%) from Central/South America and 1 patient of Chinese origin. With reference genetic background, karyotype was retrievable for 451 out of 481 individuals, with the following distribution: 431 (96%) free trisomy of chromosome 21, 10 (2%) mosaicism and the remaining 10 (2%) Robertsonian translocation. As for comorbidities, 197 DS individuals were born with a non-cyanotic congenital heart defect (41%), 86 (18%) were found to have a thyroid disorder (including autoimmune conditions), 56 (11%) were diagnosed with celiac disease, 18 (3.7%) had a gastrointestinal defect, 10 (2%) with epilepsy, 4 (0.8%) with alopecia, and 2 (0.4%) with diabetes mellitus type 1.

### Hematological parameters: normal distribution of values in the study population

**Figure 1:**
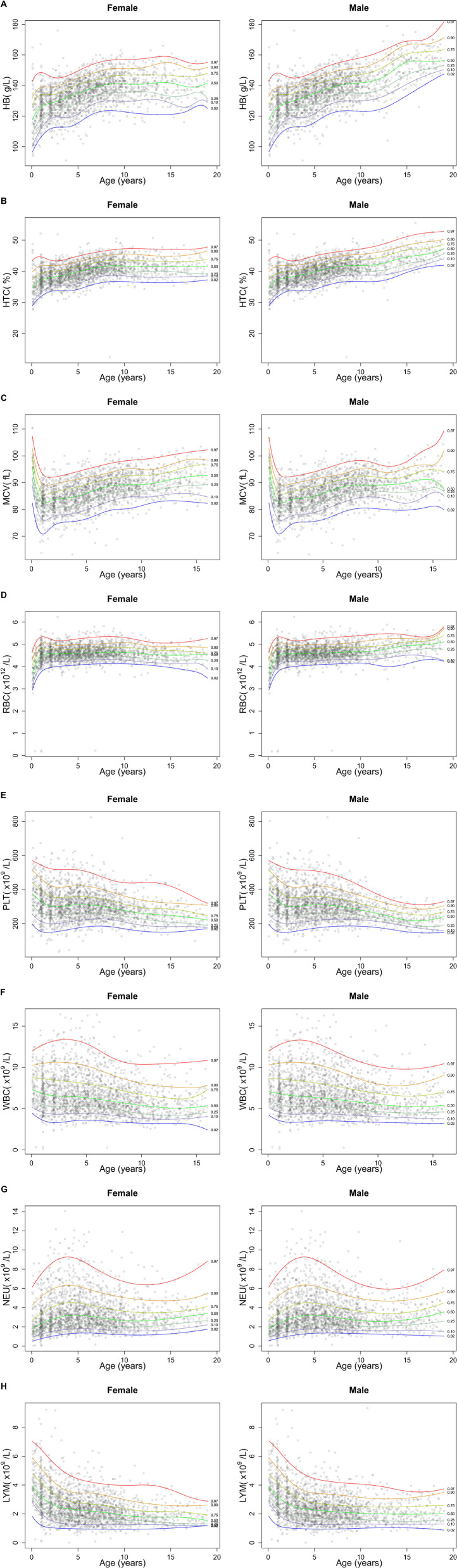
Graphical representation of centiles of CBC parameters in the study cohort of children and adolescents with DS. The figure shows the centile distribution for hemoglobin (HB, panel **A**), hematocrit (HTC, panel **B**), mean corpuscular volume (MCV, panel **C**), red blood cells (RBC, panel **D**), platelets (PLT, panel **E**), white blood cells (WBC, panel **F**), neutrophils (NEU, panel **G**) and lymphocyte (LYM, panel **H**).

Out of the 2,627 CBCs recorded, 1,062 were assessed by the Sysmex analyzer, and 1,565 with Beckman Coulter. The comparison by a non-parametric regression model using cubic splines demonstrated superimposable results, with satisfactory degree of agreement (Supplementary Figure S1). Figure 1 reports a graphical representation of the normal distribution of CBC parameters in the study population. The sex-specific nomograms display the 2.5th, 10th, 25th, 50th, 75th, 90th, and 97.5th percentiles of the recorded age-specific data. Finally, Table 1 presents percentiles for each parameter of CBCs in DS, categorized by age groups. The last two age groups (10–12 years and 13–18 years), corresponding to the age ranges in which the influence of pubertal development is expected, are further differentiated based on sex.

**Table 1:** Percentiles of the distribution of CBC parameters in the study population of Down syndrome individuals, declined by age class.

CBC parameters	Age groups	*N*	2.5°	10°	25°	50°	75°	90°	97.5°
Red blood cells ×10^12^/L	1–3 months	44	3.1	3.1	3.4	3.7	4.1	4.6	4.8
	4–12 months	108	3.7	3.9	4.2	4.4	4.7	4.9	5.2
	1–2 years	444	3.9	4.2	4.3	4.5	4.8	5.0	5.3
	3–4 years	441	4.0	4.2	4.4	4.6	4.8	5.0	5.2
	5–6 years	410	4.1	4.3	4.4	4.6	4.8	5.0	5.3
	7–9 years	463	4.2	4.3	4.5	4.7	4.9	5.1	5.3
	10–12 years, female	181	4.1	4.2	4.4	4.6	4.8	5.0	5.2
	10–12 years, male	161	4.1	4.3	4.5	4.7	4.9	5.2	5.4
	13–18 years, female	180	3.9	4.2	4.3	4.5	4.7	4.9	5.1
	13–18 years, male	195	4.3	4.5	4.7	5.0	5.1	5.3	5.5
Hemoglobin g/L	1–3 months	44	95	101	108	117	134	144	154
	4–12 months	108	102	109	117	124	132	136	142
	1–2 years	444	111	116	122	128	134	139	146
	3–4 years	441	113	120	126	132	138	143	146
	5–6 years	410	120	125	132	137	142	146	153
	7–9 years	463	124	130	135	141	146	151	156
	10–12 years, female	181	120	130	136	140	147	151	156
	10–12 years, male	161	123	131	136	143	149	154	160
	13–18 years, female	180	126	129	135	142	146	153	158
	13–18 years, male	195	136	142	149	155	160	166	170
Hematocrit %	1–3 months	44	28.0	29.7	32.5	35.0	38.2	43.4	46.8
	4–12 months	108	30.9	32.8	34.8	36.6	38.4	41.1	42.5
	1–2 years	444	33.5	34.7	36.1	37.9	39.9	41.8	43.3
	3–4 years	441	33.6	35.6	37.2	38.9	40.6	42.4	43.8
	5–6 years	410	35.3	37.1	38.7	40.4	42.0	44.0	45.6
	7–9 years	463	36.6	38.6	40.0	41.6	43.4	45.1	46.7
	10–12 years, female	181	36.3	37.9	39.5	41.3	43.2	45.0	46.6
	10–12 years, male	161	36.5	38.6	40.1	41.9	44.0	45.6	47.3
	13–18 years, female	180	36.6	38.7	40.0	41.6	43.1	45.5	47.2
	13–18 years, male	195	39.5	41.2	43.0	44.8	46.9	48.5	51.5
Mean corpuscular volume fl	1–3 months	44	85.0	86.8	89.8	95.3	98.4	100.6	110.0
	4–12 months	108	72.0	76.2	81.0	83.4	86.2	89.4	92.9
	1–2 years	444	73.5	77.5	80.5	83.6	86.5	89.4	92.8
	3–4 years	441	75.5	79.2	81.9	85.1	88.3	91.2	93.8
	5–6 years	410	77.6	80.9	84.1	87.4	90.5	92.6	95.6
	7–9 years	463	80.3	84.3	86.5	89.0	92.2	94.9	98.2
	10–12 years, female	181	83.2	84.7	87.4	90.2	93.3	95.2	99.2
	10–12 years, male	161	79.9	83.2	87.0	89.9	91.9	94.0	96.1
	13–18 years, female	180	82.5	85.7	89.0	92.1	95.8	98.7	103.5
	13–18 years, male	195	80.8	85.2	88.2	91.1	94.5	98.7	103.1
White blood cells ×10^9^/L	1–3 months	44	5.2	5.6	6.2	7.5	8.7	11.1	12.1
	4–12 months	108	3.7	4.9	5.7	6.8	9.3	10.6	12.6
	1–2 years	444	3.3	4.3	5.2	6.6	8.3	10.4	13.2
	3–4 years	441	3.5	4.4	5.2	6.4	8.0	10.0	12.8
	5–6 years	410	3.6	4.3	5.2	6.3	8.0	10.2	12.2
	7–9 years	463	3.3	4.1	4.8	5.8	7.1	8.4	10.7
	10–12 years, female	181	3.1	3.7	4.1	5.1	6.3	7.8	10.2
	10–12 years, male	161	3.4	4.1	4.6	5.2	6.5	7.7	10.1
	13–18 years, female	180	3.2	3.9	4.4	5.4	6.4	7.4	10.1
	13–18 years, male	195	3.2	3.8	4.6	5.8	7.0	8.1	9.6
Lymphocyte absolute count ×10^9^/L	1–3 months	44	2.8	2.9	3.6	4.0	4.8	5.5	6.1
	4–12 months	108	2.0	2.4	2.8	3.6	4.7	6.3	6.9
	1–2 years	444	1.0	1.6	2.1	2.8	3.6	4.5	5.6
	3–4 years	440	1.0	1.3	1.7	2.3	2.9	3.6	4.8
	5–6 years	408	1.0	1.3	1.7	2.2	2.7	3.5	4.5
	7–9 years	462	1.0	1.2	1.6	2.1	2.6	3.2	3.9
	10–12 years, female	181	0.9	1.2	1.5	1.8	2.2	2.7	4.1
	10–12 years, male	161	1.1	1.4	1.6	2.0	2.5	3.2	4.2
	13–18 years, female	178	1.0	1.2	1.4	1.8	2.1	2.6	3.3
	13–18 years, male	195	1.0	1.3	1.5	2.0	2.5	3.1	3.5
Neutrophil absolute count ×10^9^/L	1–3 months	44	0.6	0.9	1.4	1.9	2.8	5.2	7.3
	4–12 months	108	0.7	1.2	1.6	2.1	2.8	4.4	7.0
	1–2 years	444	0.9	1.5	1.9	2.7	3.9	5.8	8.0
	3–4 years	440	1.3	1.8	2.3	3.2	4.4	6.2	9.1
	5–6 years	408	1.3	1.8	2.3	3.2	4.4	6.2	8.9
	7–9 years	462	1.3	1.7	2.2	2.8	3.9	5.3	7.0
	10–12 years, female	181	1.1	1.5	2.0	2.5	3.5	4.9	6.3
	10–12 years, male	161	1.3	1.6	2.0	2.5	3.4	4.4	6.2
	13–18 years, female	178	1.3	1.7	2.3	2.9	3.7	4.8	6.8
	13–18 years, male	195	1.1	1.6	2.2	2.9	3.8	5.0	6.3
Platelets ×10^9^/L	1–3 months	44	190.2	259.8	303.8	365	444.8	531.9	637.4
	4–12 months	108	160.0	216.8	280.0	359	411.2	486.0	580.6
	1–2 years	444	159.2	200.0	254.0	310	366.2	419.4	516.8
	3–4 years	441	165.0	212.0	255.0	302	353.0	423.0	515.0
	5–6 years	410	187.7	221.0	260.2	305	359.8	420.1	494.6
	7–9 years	463	186.2	223.0	255.5	293	334.0	385.4	458.8
	10–12 years, female	181	161.5	188.0	228.0	266	299.0	340.0	421.0
	10–12 years, male	161	179.0	211.0	234.0	265	303.0	334.0	375.0
	13–18 years, female	180	158.8	181.9	206.5	243	279.8	312.0	352.1
	13–18 years, male	195	147.6	175.4	199.5	224	257.0	291.6	327.9

### Online informatic tool to assess CBC's parameters centile

We developed a computational web tool aimed at verifying whether hemogram parameters fall within newly defined normal ranges for individuals with DS. By filling in the blanks, the tool provides the calculated centile for each determination with reference to our data and graphically plots up to 3 sequentially assessed values. The tool is available online at: https://b4-uni25-5627493duksfy852qr80fewbsn3986g43jkgkzie8.shinyapps.io/HematologyReferenceTrisomy21/.

### Comparison of CBC parameters in Down syndrome vs. controls

**Figure 2:**
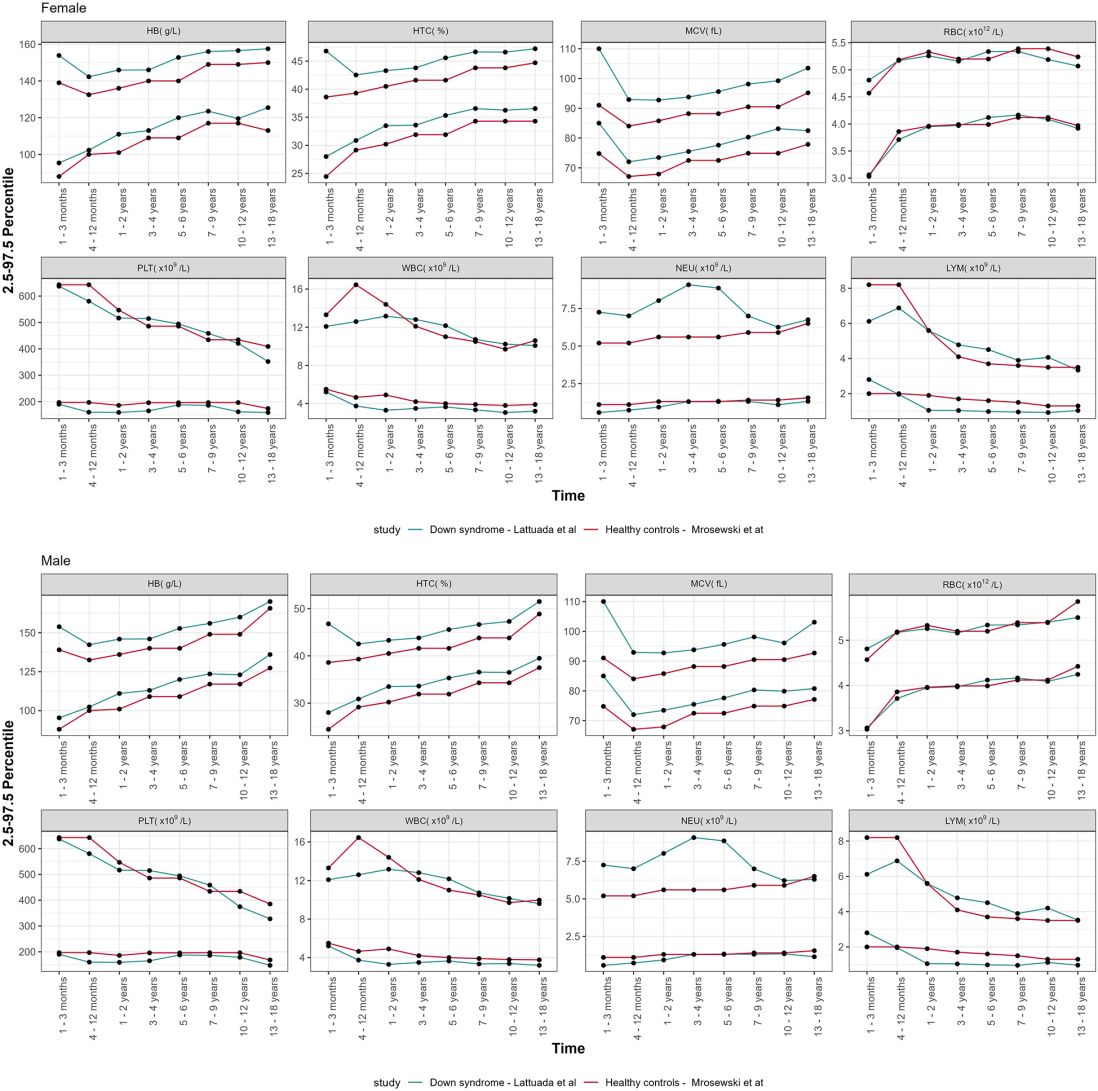
Comparison of the centile distribution of CBC parameters vs. healthy controls from the literature, declined by age groups and sexes.

The lower and upper limit of the new defined reference ranges (percentiles 2.5 and 97.5) of CBC parameters, categorized by age groups and sex, were compared with those of the general pediatric population (21), as depicted in Figure 2. The proportion of HB, HCT, and MCV values above the 97.5th centile in the study population was statistically greater than in the general pediatric population, as reported in Table 2. A statistically significant proportion of WBC values were below the 2.5th centile reported in DS population (*p* < 0.001), except for the age group 31 days–1 year. The same result was observed for LYM (*p* < 0.001), from 1 year to 18 years old. The proportion of NEU falling above the 97.5th centile or below the 2.5th centile was statistically greater and lower, respectively, over the timespan assessed (see Table 2 for additional details). Considering PTL, the proportion of values below the 2.5th centile for healthy children achieved statistical significance only for some of the age classes assessed. Conversely, we found no significant differences concerning the upper PTL limit between our DS groups and healthy controls.

**Table 2:** Comparison of reference ranges of our patients with DS and healthy controls using one-sample test on proportion.

CBC parameters	Age groups	Healty controls	DS patients			*p* Value	Healthy controls	DS patients		*p* Value
		2.5th centile	N of measures	N below 2.5th centile	Proportion of measures below the 2.5th centile		97.5th centile	N upper 97.5th centile	Proportion of measures upper the 97.5th centile	
RBC (×10^12^/L)	1–3 months	3.1	44	1	0.023	0.50	4.6	5	0.114	<0.001
	4–12 months	3.9	108	5	0.046	0.13	5.2	2	0.19	0.55
	1–2 years	3.9	444	12	0.027	0.45	5.3	5	0.011	0.96
	3–4 years	4.0	441	13	0.029	0.33	5.2	8	0.018	0.78
	5–6 years	4.0	410	4	0.010	0.97	5.2	14	0.034	0.15
	7–9 years	4.1	463	9	0.019	0.73	5.4	10	0.022	0.63
	10–12 years, female	4.1	181	8	0.044	0.08	5.4	2	0.011	0.83
	10–12 years, male	4.12	161	6	0.037	0.23	5.4	5	0.031	0.41
	13–18 years, female	4.0	180	6	0.033	0.32	5.2	1	0.006	0.93
	13–18 years, male	4.4	195	17	0.087	<0.001	5.8	0	0.000	0.98
HB (g/L)	1–3 months	88	44	0	0.000	0.72	139	9	0.205	<0.001
	4–12 months	100	108	0	0.000	0.91	133	24	0.222	<0.001
	1–2 years	101	444	0	0.000	0.99	136	79	0.178	<0.001
	3–4 years	109	441	3	0.007	0.98	140	73	0.166	<0.001
	5–6 years	109	410	5	0.012	0.93	140	137	0.334	<0.001
	7–9 years	117	463	1	0.002	0.99	149	58	0.125	<0.001
	10–12 years, female	117	181	2	0.011	0.83	149	28	0.155	<0.001
	10–12 years, male	117	161	1	0.006	0.99	149	32	0.199	<0.001
	13–18 years, female	113	180	0	0.006	0.97	150	26	0.144	<0.001
	13–18 years, male	127	195	1	0.005	0.94	166	25	0.128	<0.001
HTC (%)	1–3 months	24.4	44	0	0.000	0.72	38.6	10	0.22	<0.001
	4–12 months	29.2	108	0	0.000	0.91	39.3	19	0.17	<0.001
	1–2 years	30.2	444	0	0.000	0.99	40.5	79	0.17	<0.001
	3–4 years	31.9	441	2	0.005	0.99	41.6	66	0.15	<0.001
	5–6 years	31.9	410	1	0.002	0.99	41.6	123	0.30	<0.001
	7–9 years	34.3	463	1	0.002	0.99	43.8	96	0.20	<0.001
	10–12 years, female	34.3	181	0	0.000	0.97	43.8	32	0.17	<0.001
	10–12 years, male	34.3	161	2	0.012	0.77	43.8	44	0.273	<0.001
	13–18 years, female	34.3	180	0	0.000	0.97	44.7	22	0.12	<0.001
	13–18 years, male	37.5	195	0	0.000	0.97	48.9	18	0.092	<0.001
MCV (fl)	1–3 months	74.8	44	0	0.000	0.72	91.0	31	0.705	<0.001
	4–12 months	67.1	108	1	0.009	0.77	84.0	47	0.435	<0.001
	1–2 years	67.9	444	0	0.000	0.99	85.8	129	0.291	<0.001
	3–4 years	72.5	441	1	0.002	0.99	88.2	111	0.252	<0.001
	5–6 years	72.5	410	3	0.007	0.98	88.2	172	0.420	<0.001
	7–9 years	74.9	463	1	0.002	0.99	90.5	170	0.367	<0.001
	10–12 years, female	74.9	181	0	0.000	0.97	90.5	83	0.459	<0.001
	10–12 years, male	74.9	161	0	0.000	0.96	90.5	65	0.404	<0.001
	13–18 years, female	77.9	180	0	0.000	0.97	95.2	49	0.272	<0.001
	13–18 years, male	77.1	195	0	0.000	0.98	92.7	78	0.400	<0.001
WBC (×10^9^/L)	1–3 months	5.5	44	3	0.068	0.1	13.3	0	0.000	0.72
	4–12 months	4.6	108	8	0.074	<0.001	16.5	0	0.000	0.91
	1–2 years	4.9	444	85	0.191	<0.001	14.4	2	0.005	0.99
	3–4 years	4.2	441	33	0.075	<0.001	12.1	15	0.034	0.15
	5–6 years	4.0	410	21	0.051	<0.001	11.0	26	0.063	<0.001
	7–9 years	3.9	463	34	0.073	<0.001	10.5	15	0.032	0.19
	10–12 years, female	3.8	181	27	0.149	<0.001	9.7	7	0.039	0.17
	10–12 years, male	3.8	161	8	0.050	0.04	9.7	7	0.043	0.11
	13–18 years, female	3.9	180	19	0.106	<0.001	10.6	4	0.022	0.50
	13–18 years, male	3.8	195	16	0.082	<0.001	9.9	5	0.026	0.50
LYM (×10^9^/L)	1–3 months	2.0	44	0	0.000	0.72	8.2	0	0.000	0.72
	4–12 months	2.0	108	4	0.037	0.31	8.2	2	0.019	0.55
	1–2 years	1.9	444	78	0.176	<0.001	5.6	11	0.025	0.50
	3–4 years	1.7	441	108	0.245	<0.001	4.1	26	0.059	<0.001
	5–6 years	1.6	410	88	0.215	<0.001	3.7	28	0.068	<0.001
	7–9 years	1.5	463	94	0.203	<0.001	3.6	24	0.052	<0.001
	10–12 years, female	1.3	181	24	0.133	<0.001	3.5	6	0.033	0.32
	10–12 years, male	1.3	161	14	0.087	<0.001	3.5	7	0.036	0.228
	13–18 years, female	1.3	180	28	0.156	<0.001	3.5	4	0.002	0.50
	13–18 years, male	1.3	195	24	0.123	<0.001	3.5	7	0.036	0.23
NEU (×10^9^/L)	1–3 months	1.1	44	6	0.136	<0.001	5.2	5	0.114	<0.001
	4–12 months	1.1	108	10	0.093	<0.001	5.2	9	0.083	<0.001
	1–2 years	1.3	444	35	0.079	<0.001	5.6	48	0.108	<0.001
	3–4 years	1.3	441	11	0.025	0.50	5.6	58	0.132	<0.001
	5–6 years	1.3	410	9	0.022	0.59	5.6	61	0.149	<0.001
	7–9 years	1.4	463	19	0.041	0.02	5.9	27	0.058	<0.001
	10–12 years, female	1.4	181	13	0.072	<0.001	5.9	8	0.044	0.08
	10–12 years, male	1.4	161	5	0.031	0.41	5.9	7	0.043	0.11
	13–18 years, female	1.55	180	8	0.044	0.08	6.5	5	0.028	0.50
	13–18 years, male	1.55	195	17	0.087	<0.001	6.5	3	0.015	0.74
PLT (×10^9^/L)	1–3 months	197.0	44	3	0.068	0.09	643.0	2	0.045	0.35
	4–12 months	197.0	108	8	0.074	<0.001	643.0	1	0.009	0.77
	1–2 years	186.0	444	31	0.070	<0.001	547.0	7	0.016	0.86
	3–4 years	196.0	441	29	0.066	<0.001	486.0	17	0.039	0.05
	5–6 years	196.0	410	14	0.034	0.15	486.0	14	0.034	0.15
	7–9 years	196.5	463	17	0.037	0.07	434.5	18	0.039	0.04
	10–12 years, female	196.5	181	20	0.110	<0.001	434.5	4	0.022	0.51
	10–12 years, male	196.5	161	9	0.056	0.01	434.5	2	0.012	0.78
	13–18 years, female	174.0	180	11	0.061	<0.001	409.0	4	0.022	0.50
	13–18 years, male	168.5	195	15	0.077	<0.001	385.5	0	0.000	0.98

**Figure 3:**
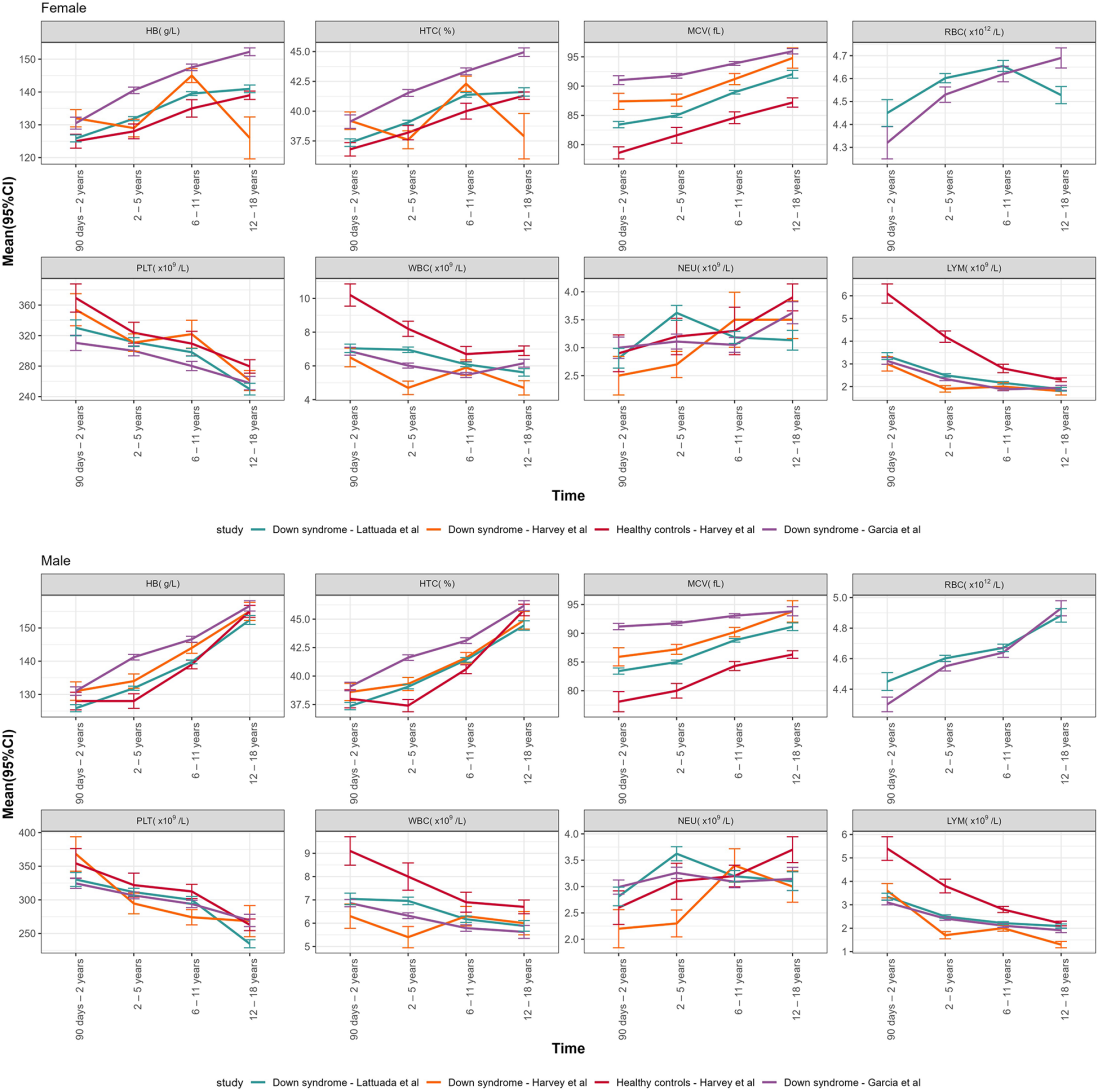
Graphical representation of the mean (95% CI) of CBC DS parameters of our study vs. Garcia's DS population, Harvey's DS population and Harvey's healthy controls, declined by age groups and sexes.

Figure 3 summarizes the comparison between our data and two recent studies conducted on children with DS (18, 19). The mean and SD for all our parameters, collected in four age groups (90 days–2 years, 2–5 years, 6–1 years, 12–18 years), for both sexes, were compared with Garcia's DS population, Harvey's DS population and Harvey's healthy controls, as reported in Table 3.

**Table 3:** Comparison of the mean and standard deviation of CBC DS parameters of our study vs. Garcia's DS population, Harvey's DS population and Harvey's healthy controls.

Age groups	*N*	Mean	Sd	lowCI	uppCI	Mean Harvey	*p*-value vs. Harvey	Mean Harvey CTRL	*p*-value vs. Harvey CTRL	Mean Garcia	*p*-value vs. Garcia
Female											
Hemoglobin, g/L											
90 days–2 years	337	125.85	0.993	124.78	126.91	132	<0.001	125	0.129	130.50	<0.001
2–5 years	874	131.87	0.939	131.24	132.49	129	<0.001	128	<0.001	140.50	<0.001
6–11 years	797	139.51	0.835	138.93	140.09	145	<0.001	135	<0.001	147.50	<0.001
12–18 years	229	140.95	0.894	139.78	142.11	126	<0.001	139	0.002	152.25	<0.001
Hematocrit,%											
90 days–2 years	337	37.360	2.969	37.042	37.678	39.2	<0.001	36.8	0.002	39.12	<0.001
2–5 years	874	39.059	2.749	38.877	39.242	37.6	<0.001	38.2	<0.001	41.53	<0.001
6–11 years	797	41.369	2.835	41.172	41.566	42.3	<0.001	40.0	<0.001	43.34	<0.001
12–18 years	229	41.613	2.684	41.264	41.963	37.9	<0.001	41.3	0.116	44.95	<0.001
Mean corpuscular volume, fl											
90 days–2 years	337	83.426	5.049	82.885	83.966	87.4	<0.001	78.6	<0.001	91.020	<0.001
2–5 years	874	85.000	5.008	84.668	85.333	87.6	<0.001	81.6	<0.001	91.760	<0.001
6–11 years	797	88.959	4.440	88.650	89.267	91.2	<0.001	84.6	<0.001	93.870	<0.001
12–18 years	229	92.034	5.113	91.368	92.699	94.8	<0.001	87.2	<0.001	95.955	<0.001
Red blood cells, ×10^12^/L											
90 days–2 years	337	4.450	0.545	4.391	4.508	87.4	NA	78.6	NA	4.32	<0.001
2–5 years	874	4.602	0.302	4.582	4.622	87.6	NA	81.6	NA	4.53	<0.001
6–11 years	797	4.655	0.344	4.631	4.679	91.2	NA	84.6	NA	4.62	0.005
12–18 years	229	4.528	0.288	4.491	4.566	94.8	NA	87.2	NA	4.69	<0.001
Platelets, ×10^9^/L											
90 days–2 years	337	330.249	97.336	319.820	340.679	353.9	0.000	369.3	<0.001	310.44	0.001
2–5 years	874	311.470	87.228	305.679	317.261	310.8	0.211	323.8	<0.001	300.01	0.013
6–11 years	797	298.153	72.247	293.130	303.177	321.9	<0.001	309.5	<0.001	280.16	<0.001
12–18 years	229	249.843	58.892	242.175	257.511	261.1	<0.001	279.6	<0.001	257.73	0.002
White blood cells, ×10^9^/L											
90 days–2 years	337	7.043	2.312	6.795	7.290	6.5	0.001	10.2	<0.001	6.87	0.597
2–5 years	874	6.957	2.440	6.795	7.119	4.7	<0.001	8.2	<0.001	6.02	<0.001
6–11 years	797	6.090	1.954	5.954	6.226	5.9	0.955	6.7	<0.001	5.46	<0.001
12–18 years	229	5.616	1.703	5.394	5.838	4.7	<0.001	6.9	<0.001	6.16	<0.001
Neutrophil, ×10^9^/L											
90 days–2 years	337	2.812	1.653	2.635	2.990	2.5	0.47	2.9	<0.001	3.000	<0.001
2–5 years	871	3.623	2.010	3.489	3.757	2.7	<0.001	3.2	0.043	3.110	<0.001
6–11 years	796	3.185	1.555	3.077	3.293	3.5	<0.001	3.3	<0.001	3.050	0.176
12–18 years	227	3.134	1.354	2.957	3.311	3.5	<0.001	3.9	<0.001	3.625	<0.001
Lymphocyte, ×10^9^/L											
90 days–2 years	337	3.344	1.413	3.193	3.495	3.0	0.001	6.1	0	3.130	0.095
2–5 years	871	2.499	1.034	2.430	2.568	1.9	<0.001	4.2	0	2.340	0.018
6–11 years	796	2.165	0.826	2.107	2.222	2.0	<0.001	2.8	0	1.880	<0.001
12–18 years	227	1.886	0.638	1.802	1.969	1.8	0.760	2.3	0	1.945	0.001
Male											
Hemoglobin, g/L											
90 days–2 years	337	125.85	0.993	124.78	126.91	131	<0.001	128	<0.001	131.00	<0.001
2–5 years	874	131.87	0.939	131.24	132.49	134	<0.001	128	<0.001	141.20	<0.001
6–11 years	776	139.80	0.825	139.22	140.38	144	<0.001	139	0.019	146.60	<0.001
12–18 years	245	152.41	1.066	151.07	153.75	155	0.001	155	0.001	156.65	<0.001
Hematocrit,%											
90 days–2 years	337	37.360	2.969	37.042	37.678	38.6	<0.001	38.0	<0.001	39.08	<0.001
2–5 years	874	39.059	2.749	38.877	39.242	39.3	0.020	37.4	<0.001	41.62	<0.001
6–11 years	776	41.421	2.825	41.222	41.620	41.6	0.057	40.6	<0.001	43.11	<0.001
12–18 years	245	44.437	3.299	44.022	44.852	44.9	0.036	45.8	<0.001	46.18	<0.001
Mean corpuscular volume, fl											
90 days–2 years	337	83.426	5.049	82.885	83.966	85.9	<0.001	78.1	<0.001	91.17	<0.001
2–5 years	874	85.000	5.008	84.668	85.333	87.2	<0.001	80.0	<0.001	91.74	<0.001
6–11 years	776	88.780	4.406	88.469	89.090	90.2	<0.001	84.3	<0.001	93.05	<0.001
12–18 years	245	91.138	5.305	90.470	91.806	93.8	<0.001	86.3	<0.001	93.82	<0.001

Red blood cells, ×10^12^/L											
90 days–2 years	337	4.450	0.545	4.391	4.508	85.9	NA	78.1	NA	4.30	<0.001
2–5 years	874	4.602	0.302	4.582	4.622	87.2	NA	80.0	NA	4.55	<0.001
6–11 years	776	4.671	0.344	4.646	4.695	90.2	NA	84.3	NA	4.64	0.014
12–18 years	245	4.883	0.352	4.839	4.928	93.8	NA	86.3	NA	4.93	0.143
Platelets, ×10^9^/L											
90 days–2 years	337	330.249	97.336	319.820	340.679	368.2	<0.001	354.2	<0.001	324.43	0.499
2–5 years	874	311.470	87.228	305.679	317.261	294.6	<0.001	321.9	<0.001	306.62	0.870
6–11 years	776	299.957	71.345	294.930	304.985	274.1	<0.001	312.5	<0.001	293.89	0.642
12–18 years	245	234.702	47.410	228.736	240.668	268.3	<0.001	262.9	<0.001	269.40	<0.001
White blood cells, ×10^9^/L											
90 days–2 years	337	7.043	2.312	6.795	7.290	6.3	<0.001	9.1	<0.001	6.860	0.547
2–5 years	874	6.957	2.440	6.795	7.119	5.4	<0.001	8.0	<0.001	6.320	<0.001
6–11 years	776	6.170	1.935	6.033	6.306	6.3	<0.001	6.9	<0.001	5.790	0.005
12–18 years	245	5.883	1.821	5.654	6.113	6.0	0.024	6.7	<0.001	5.625	0.279
Neutrophil, ×10^9^/L											
90 days–2 years	337	2.812	1.653	2.635	2.990	2.2	<0.001	2.6	0.654	2.990	<0.001
2–5 years	871	3.623	2.010	3.489	3.757	2.3	<0.001	3.1	<0.001	3.260	0.277
6–11 years	775	3.195	1.531	3.087	3.303	3.4	<0.001	3.2	<0.001	3.090	0.064
12–18 years	245	3.102	1.403	2.925	3.279	3.0	0.446	3.7	<0.001	3.145	0.017
Lymphocyte, ×10^9^/L											
90 days–2 years	337	3.344	1.413	3.193	3.495	3.6	<0.001	5.4	<0.001	3.10	0.042
2–5 years	871	2.499	1.034	2.430	2.568	1.7	<0.001	3.8	<0.001	2.41	0.919
6–11 years	775	2.216	0.868	2.155	2.278	2.0	<0.001	2.8	<0.001	2.11	0.239
12–18 years	245	2.087	0.738	1.994	2.180	1.3	<0.001	2.2	0.002	1.91	0.015

Sd, standard deviation; lowCI, lower confidence interval; uppCI, upper confidence interval; CTRL, controls.

## Discussion

In DS, hematopoiesis is affected by the over-expression of several genes involved in the regulation and maturation of the hematopoietic system (3), located on chromosome 21. In addition, the occurrence rates of abnormal hematological findings can be regarded in some patients as the direct effect of clinical comorbidities, such as cyanogenic cardiac defects or infectious diseases. One of the most deeply characterized genes is *RUNX1*, a transcription factor that regulates hematopoiesis and megakaryopoiesis (23). Other genes thought to be responsible for abnormal hematopoiesis include *GATA1*, *ERG*, *ETS2*, *BACH1*, *TIAM1*, *IFNAR1*, *GART*, *SON*, *SOD1*, *HMGN1*, and *USP16* (3, 24, 25). In addition, several genes encoding interferon receptors are located on chromosome 21. Accordingly, trisomy 21 can lead to an increased expression of interferon-stimulated genes, resulting in a mild interferonopathy in the microenvironment of bone marrow, with inhibitory effects on hematopoietic precursors (26).

Along with clinical and anamnestic data, the availability of reference ranges is a pivotal element in the decision-making process that eventually leads to labelling biochemical findings as pathological (27). The reliability of reference intervals mostly depends on the homogeneity of the population from which they are drawn and avoiding selection biases is pivotal to outline reproducible results (28). Given the demonstrated specificities of hematopoiesis in individuals with DS, it is advisable to compare the CBC findings with syndrome-specific reference intervals.

As in pediatrics the integrated result of growth, immune system maturation, and pubertal attainment results in physiological changes of hematopoiesis over time, percentile plotted against age can be regarded as the most reliable representation of the continuous and dynamic modifications in CBC parameters (29). Therefore, we outlined the normal distribution of CBC parameters from 31 days to 18 years of age and reported the data drawn in our population with sex-specific nomograms.

As expected, males showed a progressive increase in red blood cell parameters from the onset of puberty onwards, as a result of the promoting action of testosterone on erythropoiesis (30).

The comparison between the distribution of CBC parameters in DS and healthy controls from the literature (21, 22) outlines several differences. With reference to red blood cells parameters, individuals with DS showed a statistically significant upward shift of the distribution of hemoglobin, mean cell volume and hematocrit compared to controls, while the distribution of the concentration of RBC did not statistically differ in the two cohorts for most of the age span included in the analysis. Finally, the qualitative evaluation of the trendlines of red blood cells parameters over time showed a similar pattern in DS compared to controls.

Considering white blood cells parameters, subjects with DS had a statistically significant downward shift of the lower percentile of white blood cells and lymphocytes. Neutrophils, instead, showed a significant upward shift of the 97.5° centile compared to controls.

The distribution of PLT appeared overall superimposable between our cohort and controls.

The demonstrated statistically significant specificities in the CBC findings among individuals with DS compared to controls was consistent with the data reported in pediatric cohorts of individuals with DS from Colorado (19) and Mexico (18). The comparison between averages in our study vs. Harvey vs. Garcia de la Puente vs. controls further highlights higher mean values of HB, HTC, MCV and lower mean values of PLT, WBC, LYM in the Down population.

Neutrophils, in our DS population, were significantly higher in the age group between 2 and 5 years compared to other studies on DS. It should be noted this is the hematological parameter that is mostly affected by common intercurrent events such as infectious events, typical of pre-school childhood; although we excluded infected patients, pre-clinical or asymptomatic pictures might have justified such discrepancy.

The differences observed among the various population groups with DS could be linked to the geographic origins of the compared DS group, characterized by different genetic, epigenetic and environmental factors (i.e., altitudes, diets). Furthermore, in African and Arab populations it's observed a higher prevalence of the genetic variant of the Duffy antigen receptor for chemokines (DARC), which results in benign ethnic neutropenia (31).

The strict eligibility criteria, the uniformity of data determined by the monocentric nature of the study, the wide sample size that included more than 2,000 CBCs and the creation of an informatic tool to determine centiles, represent the strengths of our analysis.

Conversely, potential limitations include the retrospective nature of the study and the potential role of common comorbidities in Down syndrome (i.e., autoimmune diseases) in affecting blood count parameters.

Individuals with DS frequently present with alterations in complete blood count parameters, mostly not related to underlying pathological conditions and asymptomatic. The identification of syndrome-specific reference intervals, that define the physiological distribution of CBC parameters in children with DS, can play a pivotal role in clinical practice. Reference data may provide clinicians with a practical guide, also supported by the development of a dedicated informatic tool designed to estimate the centile of each recorded value with reference to the above-mentioned syndrome-specific ranges. By providing an immediate comparison of real-life findings with syndrome-specific references, the tool may prevent clinicians from prescribing unnecessary investigations. On the other hand, it may prompt timely identification of altered values that deserve urgent hematological evaluation.

Overall, it is important to emphasize that, as with any other laboratory parameter, CBC values should always be integrated with physical examination, medical history, and eventual assessment of additional biochemical parameters.

## Data Availability

The raw data supporting the conclusions of this article will be made available by the authors, without undue reservation.
