# Supplementary material for: Reference ranges for complete blood count in children and adolescents with Down syndrome
Source: Front Pediatr. 2024 Dec 11;12:1510733. doi: 10.3389/fped.2024.1510733 (PMC11668587; doi:10.3389/fped.2024.1510733)
Supplement: Supplementary file 1 [file Datasheet1.docx]

**
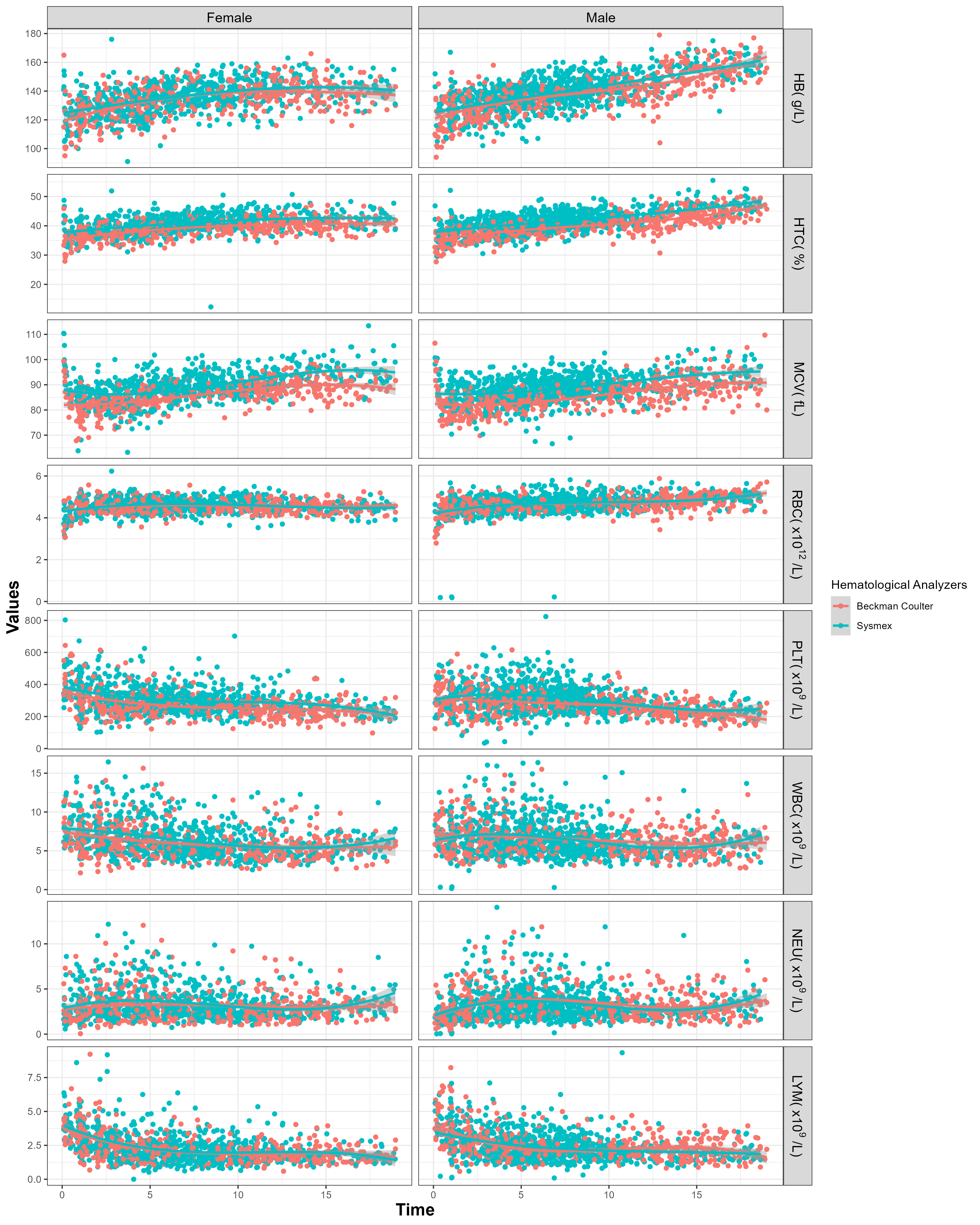
**

**Figure S1.** Comparison of the temporal trends of CBC parameters performed with Sysmex and Beckman Coulter instrumentations, using cubic splines. The x-axis shows the time expressed in life years; the y-axis shows measurement of each evaluated blood count parameter. The measurements shown in the graph, interpolated by lines, results overall aligned and superimposable.
